# The Role of TLR7 and TLR9 in the Pathogenesis of Systemic Sclerosis

**DOI:** 10.3390/ijms25116133

**Published:** 2024-06-01

**Authors:** Chenyang Wang, Kyosuke Oishi, Tadahiro Kobayashi, Ko Fujii, Motoki Horii, Natsumi Fushida, Tasuku Kitano, Shintaro Maeda, Yuichi Ikawa, Akito Komuro, Yasuhito Hamaguchi, Takashi Matsushita

**Affiliations:** 1 Department of Dermatology, Kanazawa University Graduate School of Medical Sciences, Kanazawa 920-8641, Japan; wangchenyang0302@stu.kanazawa-u.ac.jp (C.W.);; 2 Department of Plastic Surgery, Kanazawa University Hospital, Kanazawa 920-8641, Japan

**Keywords:** TLR7, TLR9, systemic sclerosis, Toll-like receptors, plasmacytoid dendritic cells, pDCs, B cells, Regulatory B cells, Effector B cells, Treg, Th17

## Abstract

The bleomycin-induced scleroderma model is a well-established and dependable method for creating a mouse model of SSc (systemic sclerosis). In the field of skin connective tissue diseases, increasing evidence from clinical and animal experiments suggests that TLRs (Toll-like receptors) play an important role in several diseases. This study aimed to determine the role of TLR7 (Toll-like receptor 7) and TLR9 (Toll-like receptor 9) in the mechanisms of immune abnormalities and fibrosis in SSc. This study used TLR7-KO mice (TLR7-knockout mice with a balb/c background) and TLR9-KO mice (TLR9-knockout mice with a balb/c background) as well as WT mice (wild-type balb/c mice). All three kinds of mice were induced by BLM (bleomycin) in a scleroderma model as the experimental group; meanwhile, WT mice treated with PBS (phosphate-buffered saline) were used as the control group. We analyzed the fibrotic phenotype and the immunological abnormality phenotype of TLR7-deficient and TLR9-deficient mice in the SSc disease model using flow cytometry, RT-PCR (reverse transcription–polymerase chain reaction), a histological examination, and IHC (immunohistochemical staining). In a mouse model of SSc disease, the deletion of TLR7 attenuated skin and lung fibrosis, while the deletion of TLR9 exacerbated skin and lung fibrosis. The deletion of TLR7 resulted in a relative decrease in the infiltration and expression of various pro-inflammatory and fibrotic cells and cytokines in the skin. On the other hand, the deletion of TLR9 resulted in a relative increase in the infiltration and expression of various pro-inflammatory and cytokine-inhibiting cells and cytokines in the skin. Under the influence of pDCs (plasmacytoid dendritic cells), the balances of Beff/Breg (IL-6 + CD19 + B cell/IL-10 + CD19 + B cell), Th17/Treg (IL-17A + CD4 + T cell/Foxp3 + CD25 + CD4 + T cell), M1/M2 (CD86 + macrophage/CD206 + macrophage), and Th1/Th2 (TNFα + CD3 + CD4 + T cell/IL-4 + CD3 + CD4 + T cell) were biased towards the suppression of inflammation and fibrosis as a result of the TLR7 deletion. Comparatively, the balance was biased towards promoting inflammation and fibrosis due to the TLR9 deletion. In the SSc model, TLR7 promoted inflammation and fibrosis progression, while TLR9 played a protective role. These results suggest that TLR7 and TLR9 play opposite roles in triggering SSc to produce immune system abnormalities and skin fibrosis.

## 1. Introduction

SSc is an autoimmune disease characterized by vascular damage and fibrosis of the skin and internal organs [1]. Previous studies have shown that complex interactions between B lymphocytes, T lymphocytes, macrophages, dendritic cells, and fibroblasts lead to fibroblast activation and the excessive production and accumulation of the extracellular matrix [2]. Interactions between cells are mediated by various cytokines using molecules such as chemokines [3,4]. In animal SSc models, various cytokines, chemokines, lymphocytes, and monocytes show elevated expression levels. In cellular experiments related to SSc, the imbalance of different cells and cytokines directly affects fibrosis, either promoting or inhibiting it [5,6,7].

In SSc, the depletion of splenic pDCs contributes to the attenuation of skin fibrosis [8,9]. Additionally, both ssDNA (single-stranded DNA) and dsDNA (double-stranded DNA) are involved in the pathogenesis of SSc [10]. Furthermore, persistent inflammation is associated with the progression of fibrosis in SSc [10]. In SLE (systemic lupus erythematosus), TLR7 and TLR9 have opposing effects on disease progression [11,12]. The balance of Beff/Breg, Th1/Th2, and Th17/Treg have opposite effects on SLE and SSc [13,14,15].

TLR7 and TLR9 are PRRs (pattern recognition receptors) that recognize PAMPs (pathogen-associated molecular patterns) and DAMPs (damage-associated molecular patterns). The activation of TLR7 and TLR9 in SSc and SLE leads to the overproduction of IFNα. In SSc and SLE, both pro-inflammatory cytokines and inflammation-inhibiting cells and cytokines are disrupted. This similarity suggests that, despite the differences in clinical manifestations between SSc and SLE, they share a common pathway of immune hyperactivation and autoantibody production in their pathomechanisms [14,16,17,18,19].

## 2. Results

### 2.1. Skin and Lung Fibrosis Decreased in TLR7-KO Mice and Increased Significantly in TLR9-KO Mice

**Figure 1:**
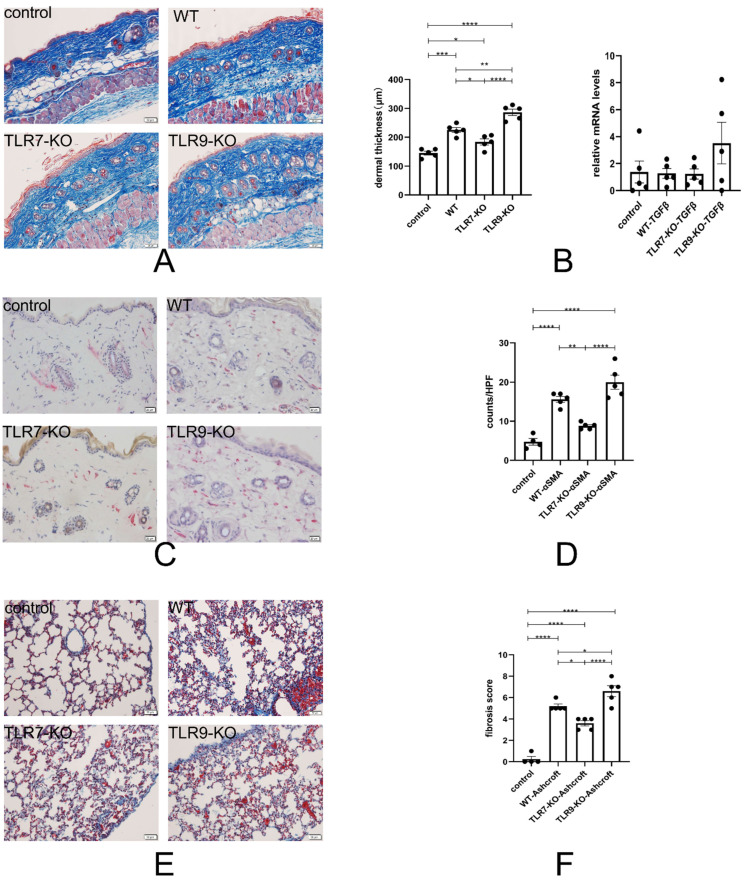
(**A**) Examples of Masson’s trichrome-stained skin tissue sections. The scale bars are all 50 μm. The dermal thicknesses in the blue sections were all measured at the same location, as indicated by the red scale bar. (**B**) The left panel corresponds to a bar chart of the dermal thickness of each mouse skin. The right panel shows the mRNA expression of TGFβ in the skin. (**C**) Immunohistochemical sections of αSMA-positive cell infiltration in the skin. The red part is αSMA-positive cells. The scale bars are all 20 μm. (**D**) Bar graphs of each type of mouse, corresponding to the C graph. (**E**): Example of a section of mouse lung tissue stained with Masson’s trichrome. All are microscopic images of the same site in the lower lateral part of the left lung. The scale bars are all 50 μm. (**F**) The degree of fibrosis in the (**E**) was quantified by using the Ashcroft Score and made into a bar chart. * = *p* < 0.05, ** = *p* < 0.01, *** = *p* < 0.001, and **** = *p* < 0.0001 for each type of mouse in each type of experiment, with a sample size of 4–5 mice. The bar denotes the mean SEM. The name of each type of mouse is shown in the upper left-hand corner of each example figure.

To evaluate the effects of separate deletions of TLR7 and TLR9 on dermal fibrosis, we evaluated skin sections from mice treated with PBS and BLM for 4 weeks. Figure 1A,B show a decrease in the skin thickness in BLM-treated TLR7-KO mice compared to BLM-treated WT mice. In contrast, an increase in the thickness was observed in BLM-treated TLR9-KO mice. Furthermore, in the mRNA expression of mouse skin tissues, the transforming growth factor β (TGFβ) expression levels were higher in BLM-treated TLR9-KO mice compared to WT and TLR7-KO mice. Figure 1C,D demonstrate that, after the BLM injection, the infiltration of αSMA-positive cells in the dermis decreased in TLR7-KO mice compared to WT mice, while it was increased in TLR9-KO mice. The BLM-induced mouse model presented with skin sclerosis and pulmonary fibrosis, as shown in Figure 1E,F. The histological analysis revealed that TLR7-KO mice treated with bleomycin had less lung fibrosis than BLM-treated WT mice, whereas TLR9-KO mice treated with bleomycin had more lung fibrosis than BLM-treated WT mice. The lung fibrosis results in these mice were consistent with those in mouse skin. These results suggest that TLR7 and TLR9 are a pair of genes that are critical for regulating skin and lung fibrosis in the BLM-induced SSc model, and that both play opposite roles.

### 2.2. A TLR7 Deletion Had a Greater Effect on Altering the Proportion of pDCs in the Spleen Than a TLR9 Deletion

**Figure 2:**
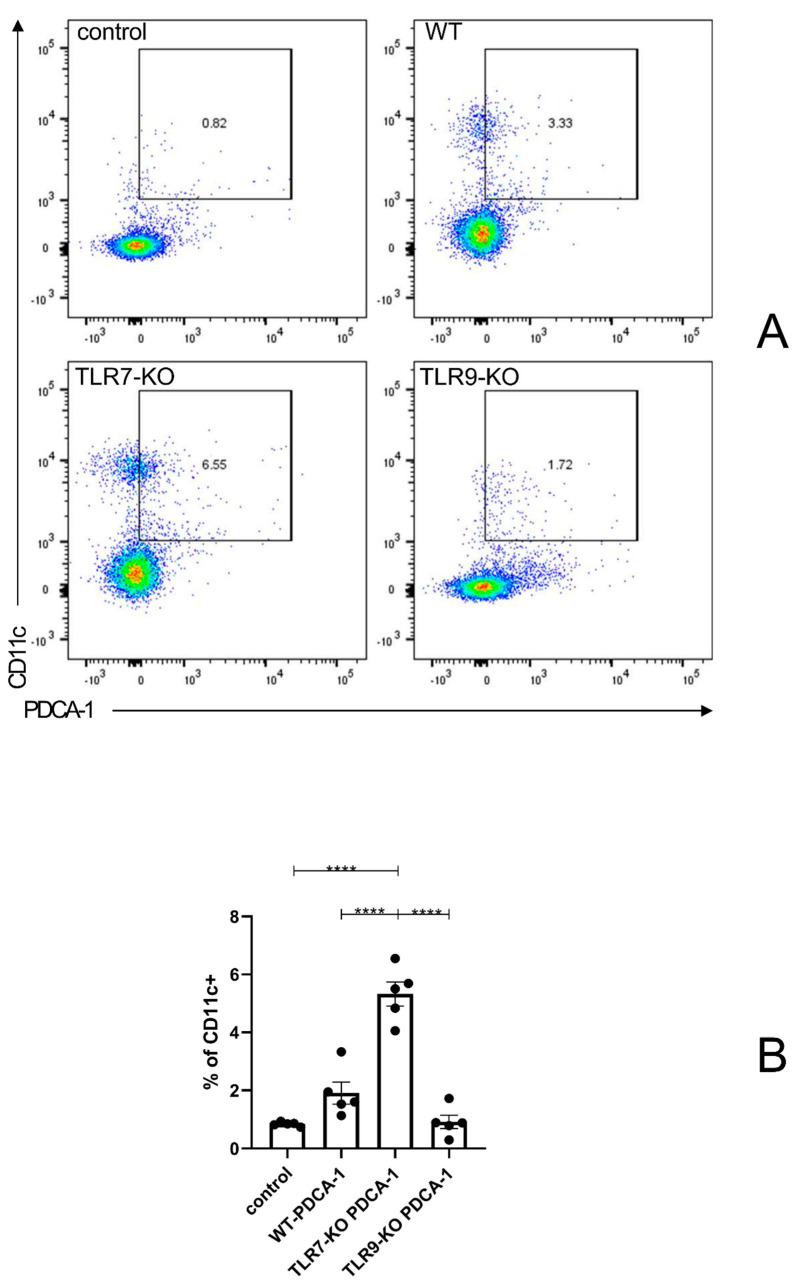
(**A**) Flow cytometry results from an independent experiment, reflecting the proportion of CD11c-staining-positive cells with positive PDCA-1 staining. (**B**) Bar graphs for each mouse species, corresponding to the left panel. **** = *p* < 0.0001 for each type of mouse in each type of experiment, with a sample size of 4–5 mice. The bar denotes the mean SEM. The name of each type of mouse is shown in the upper left corner of each example graph.

After analyzing the flow cytometry results, we observed the phenotypic characteristics of pDCs in the spleens of mice after the deletion of TLR7 or TLR9 alone. Figure 2A,B show that, after the BLM treatment, the proportion of pDCs in the TLR7-KO mice was significantly enhanced compared to the proportions in the BLM-treated WT mice. While a decrease in the proportion of pDCs was observed in TLR9-KO mice compared to BLM-treated WT mice, it was not statistically significant. In conclusion, the elevated pDCs ratio caused by the TLR7 deletion is the key to indirectly inhibiting tissue fibrosis in skin and lung tissue. The evidence suggests that a TLR9 deletion has little effect on the proportion of pDCs, indicating that a TLR9 deletion is not a key factor in increasing the degree of skin and lung fibrosis. 

### 2.3. The TLR7-KO Enhanced the Tissue-Protective Role of Cytokine IL-10. Meanwhile, the TLR9-KO Reduced the Tissue-Damaging Role of Cytokine IL-6

The flow cytometry data on IL-10 + CD19 + B cells in the spleen and the mRNA expression of IL-10 in skin tissues showed the phenotypic characterization of B cells in the spleen and skin tissues in this disease model by deleting TLR7 or TLR9 separately. The spleens of WT mice treated with BLM showed a significant increase in the proportion of IL-10 + CD19 + B cells compared to PBS-treated WT mice. Additionally, the proportion of IL-10 + CD19 + B cells was even higher in the spleens of BLM-treated TLR7-KO mice.

**Figure 3:**
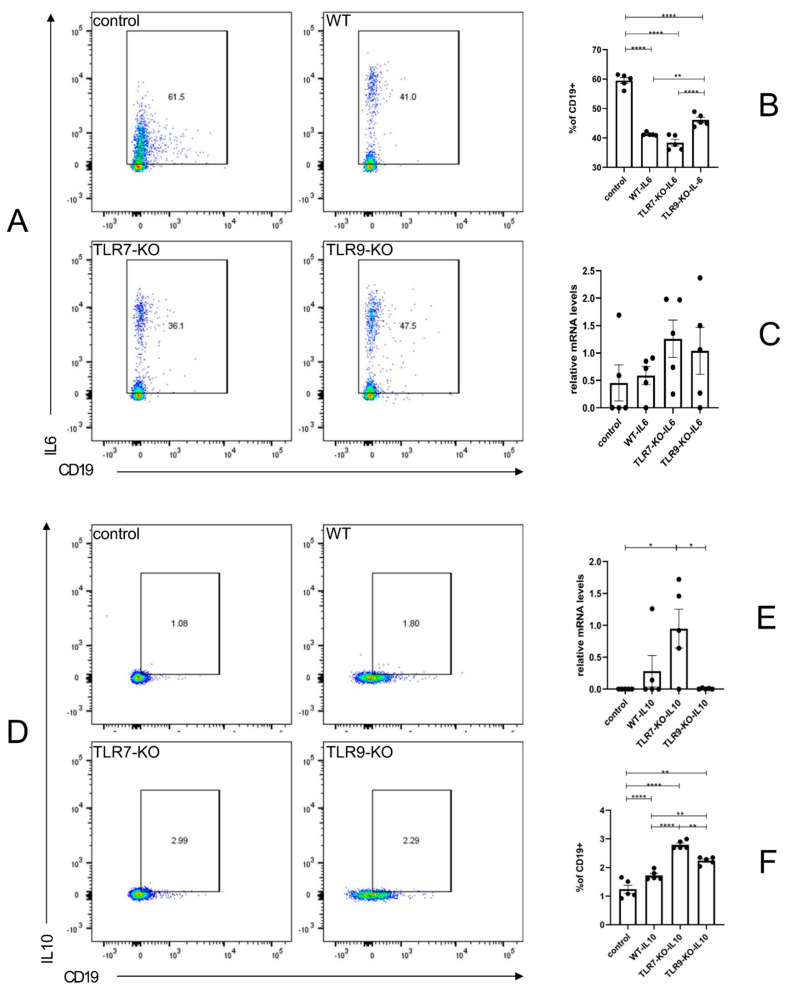
(**A**) Flow cytometry results, reflecting the proportion of cells positive for IL-6 staining in an independent experiment. (**B**) Bar graphs of CD19 + IL6 + for each mouse species, corresponding to (**A**). (**C**) The mRNA expression of IL-6 in skin tissue. (**D**) The flow cytometry results, reflecting the proportion of IL-10 staining in CD19-positive cells in an independent experiment. (**E**) The mRNA expression of IL-10 in skin tissue. (**F**) Bar graphs for each type of mouse corresponding to the graph. * = *p* < 0.05, ** = *p* < 0.01, and **** = *p* < 0.0001 for each type of mouse in each type of experiment, with sample sizes of 4–5 mice. The bar denotes the mean SEM. The name of each type of mouse is shown in the upper left corner of each example graph.

Furthermore, the proportion of IL-10 + CD19 + B cells was elevated in the spleens of BLM-treated TLR9-KO mice compared to BLM-treated WT mice. However, the increase was less significant than in BLM-treated TLR7-KO mice (Figure 3D,F). Furthermore, the mRNA expression of IL-10 was significantly increased in the skin of BLM-treated TLR7-KO mice (Figure 3E). Figure 3A,B show that the proportion of IL-6 + CD19 + B cells in the spleens of WT, TLR7-KO, and TLR9-KO mice treated with BLM was significantly lower than that of WT mice treated with PBS. However, the decrease in the IL-6 + CD19 + B cell ratio in the spleen of BLM-treated TLR9-KO mice was less significant than in BLM-treated WT and TLR7-KO mice. Figure 3C shows elevated levels of IL-6 mRNA expression in both groups, but the difference was not statistically significant. In the spleen of the BLM-induced SSc mouse model, the separate deletion of the TLR7 and TLR9 genes can independently inhibit the inflammatory response by elevating the ratio of IL-10 + CD19 + B cells. This ultimately inhibits the progression of tissue inflammation in the skin. In the spleen of the BLM-induced SSc mouse model, the deletion of either the TLR7 or TLR9 gene significantly reduced the IL-6 + CD19 + B cell ratio during an immune abnormality. This ultimately migrated to the skin tissues and promoted inflammation and fibrosis progression.

### 2.4. In the Spleen, the TLR7-KO Enhanced the Suppressive Inflammatory Effects of Foxp3 + CD25 + CD4 + Treg Cells, and the TLR9-KO Attenuated the Pro-Inflammatory Effects of IL-17A + CD4 + Treg Cells

**Figure 4:**
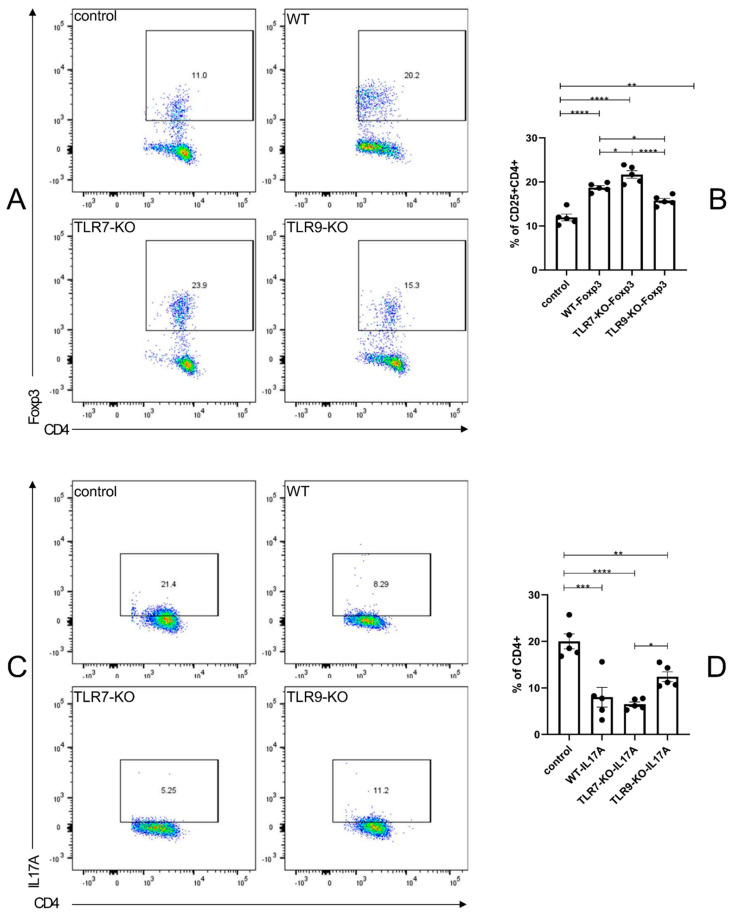
(**A**) Flow cytometry results, reflecting the proportion of cells positive for Foxp3 staining in an independent experiment with positive CD4 and CD25 staining. (**B**) Bar graphs for each mouse species, corresponding to (**A**). (**C**) The flow cytometry results, reflecting the proportion of IL-17A-positive cells in CD4-positive cells in an independent experiment. (**D**) Corresponding bar charts for each mouse species. * = *p* < 0.05, ** = *p* < 0.01, *** = *p* < 0.001, and **** = *p* < 0.0001 for each type of mouse in each type of experiment, with sample sizes of 4–5 mice. The bar denotes the mean SEM. The name of each type of mouse is shown in the upper left corner of each example graph.

The flow cytometric analysis of the splenic Foxp3 + CD25 + CD4 + Treg and IL-17A + CD4 + Th17 cells prompted the evaluation of separate TLR7 and TLR9 deletions on the immunological effects of Treg and Th17 cells in the activated state of the disease model. An increase in the proportion of Foxp3 + CD25 + CD4 + Treg cells was observed in the spleens of WT mice treated with BLM compared to PBS-treated WT mice. Additionally, there was an even greater increase in BLM-treated TLR7-KO mice. However, the proportion of Foxp3 + CD25 + CD4 + Treg cells decreased in BLM-treated TLR9-KO mice compared to BLM-treated WT mice (Figure 4A,B). Figure 4C,D show that the proportions of IL-17A + CD4 + Th17 cells in the spleens of WT, TLR7-KO, and TLR9-KO mice treated with BLM were significantly lower than those of WT mice treated with PBS. However, the proportion of Foxp3 + CD25 + CD4 + Treg cells only slightly decreased in the spleens of BLM-treated TLR9-KO mice. These results suggest that, in the spleen of the BLM-induced SSc mouse model, the TLR7 and TLR9 genes have possible indirect effects on the inflammatory state of the skin and the process of damage repair. This is achieved by promoting or suppressing the inflammatory response of CD4 + T cell subtypes with different activation states independently of each other. Thus, in the process of immune abnormalities, the inflammatory state of the skin and the process of damage repair are influenced.

### 2.5. The TLR7-KO Increased the Contribution of IL-4 + CD4 + Th2 Cells to Suppress Inflammation in the Spleen, While the TLR9-KO Increased the Contribution of TNFα + CD4 + Th1 Cells to Promote Inflammatory Processes in the Spleen

**Figure 5:**
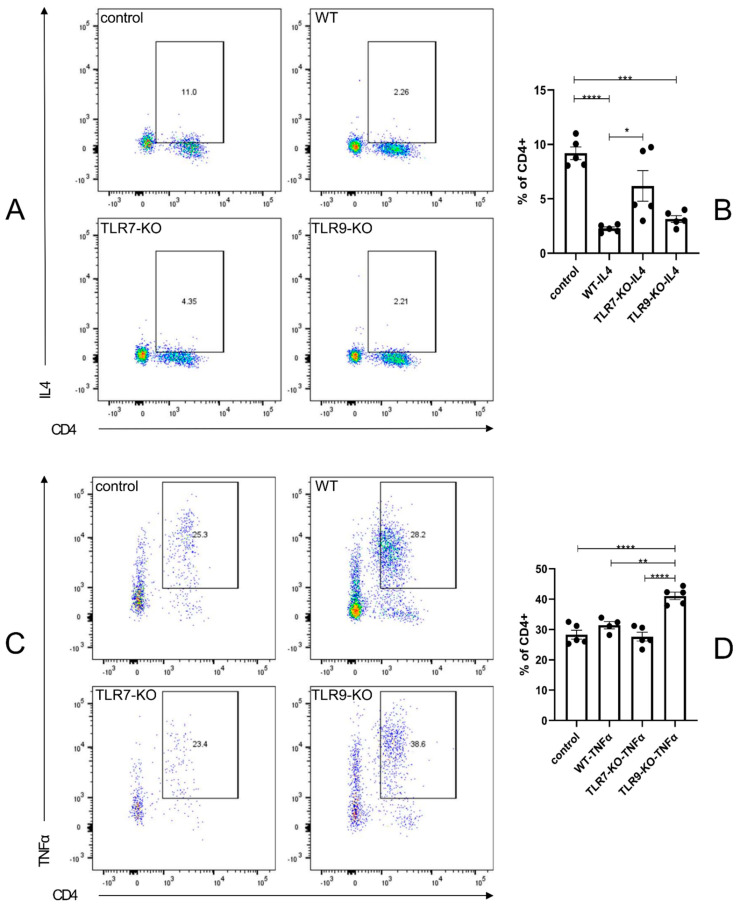
(**A**) Flow cytometry results, reflecting the proportion of cells positive for IL-4 staining in an independent experiment. (**B**) Bar charts for each mouse species, corresponding to (**A**). (**C**) The flow cytometry results, reflecting the proportion of CD4-positive cells staining positive for TNFα in an independent experiment. (**D**) Corresponding bar charts for each mouse species. * = *p* < 0.05, ** = *p* < 0.01, *** = *p* < 0.001, and **** = *p* < 0.0001 for each type of mouse in each type of experiment, with sample sizes of 4–5 mice. The bar denotes the mean SEM. The name of each type of mouse is shown in the upper left corner of each example graph.

The flow cytometric analysis results of splenic IL-4 + CD4 + Th2 and TNFα + CD4 + Th1 cells revealed that the Th1 and Th2 phenotypic characteristics of TLR7 and TLR9 were each individually and separately deficient in the activated state in the spleen of the disease model. In Figure 5A,B, we observed a significant reduction in the proportion of IL-6 + CD19 + Beff cells in the spleens of BLM-treated WT, TLR7-KO, and TLR9-KO mice compared to the control mice. However, the proportion of IL-4 + CD4 + Th2 cells in the spleens of BLM-treated TLR7-KO mice was slightly less than in BLM-treated WT and TLR9-KO mice. The spleens of TLR9-KO mice treated with BLM showed a significantly higher proportion of TNFα + CD4 + Th1 cells compared to PBS-treated WT mice, BLM-treated WT mice, and BLM-treated TLR7-KO mice. The statistical graphs indicated that the proportion of TNFα + CD4 + Th1 cells in BLM-treated TLR7-KO mice reduced more than in BLM-treated WT mice, but the difference was not statistically significant (Figure 5C,D). These results suggest that inhibiting the attenuation of skin and lung tissue fibrosis in the BLM-induced SSc mouse model was achieved by decreasing the proportion of IL-4 + CD4 + Th2 cells in the spleen through deleting TLR7. On the other hand, a TLR9 deficiency leads to fibrosis in skin and lung tissues by enhancing the expression of TNFα + CD4 + Th1 cells in the spleen.

### 2.6. The TLR9-KO Enhanced the Chronic Inflammatory Response of CD86-Positive Macrophages to the Skin, Whereas the TLR7-KO Inhibited the Chronic Inflammatory Response of CD206-Positive Macrophages to the Skin

**Figure 6:**
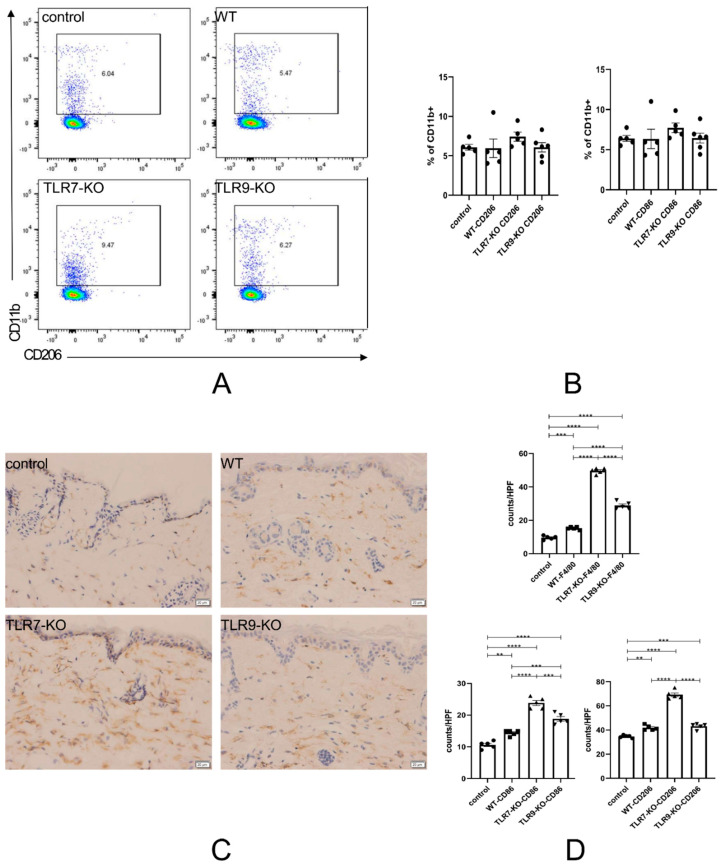
(**A**) Flow cytometry results, reflecting the proportion of CD206-positive cells in an experiment with CD11b-positive cells. (**B**) The left panel shows the bar graph corresponding to (**A**). The right panel shows the flow cytometry results, reflecting the proportion of CD86-positive cells in the same experiment. The right panel shows the bar graph corresponding to the flow cytometry results, reflecting the proportion of CD86-positive cells in the same experiment. (**C**) An example of an immunohistochemical section of skin infiltrated with F4/80-positive cells. (**D**) The upper panel is the bar chart corresponding to (**C**) for each type of mouse skin. In the lower left and lower right are bars reflecting the infiltration of CD86-positive cells and CD206-positive cells in the skin of each of the four mouse species, respectively. ** = *p* < 0.01, *** = *p* < 0.001, and **** = *p* < 0.0001 for each type of mouse in each type of experiment, with a sample size of 4–5 mice. The bar denotes the mean SEM. The name of each type of mouse is shown in the upper left corner of each example plot.

After analyzing the flow cytometry results of CD86 + CD11b + M1 macrophages and CD206 + CD11b + M2 macrophages in mouse spleens and the infiltration of F4/80-, CD86-, and CD206-positive cells in the skin, we observed a significant number of macrophage cells in splenic and skin tissues after the deletion of TLR7 and TLR9 individually. The deletion of TLR7 and TLR9 did not influence the phenotypic characteristics of the cells. Figure 6A,B show that, in the same set of experiments, the proportions of CD86 + CD11b + M1 macrophages and CD206 + CD11b + M2 macrophages in BLM-treated TLR7-KO mice were increased compared to those in BLM-treated WT and TLR9-KO mice; however, the differences were not statistically significant. The number of F4/80-positive cells in BLM-treated TLR7-KO mice was significantly higher than in BLM-treated WT mice, and the number of F4/80-positive cells infiltrated in skin tissues was also markedly higher than in BLM-treated WT mice (Figure 6C,D, upper panels).

Similarly, the number of F4/80-positive cells in BLM-treated TLR9-KO mice was higher than in WT mice, but less than BLM-treated TLR7-KO mice. The statistical graph of CD206-positive cells shows that the number of CD86-positive cells infiltrated in the skin of BLM-treated TLR9-KO mice was higher than the number of CD206-positive cells infiltrated in the skin of WT mice, and this difference is statistically significant (Figure 6D, lower panel). However, there was no significant difference in the number of CD206-positive cells infiltrating the skin of BLM-treated WT mice and TLR9-KO mice, as shown by the statistical plots reflecting the infiltration of CD206-positive cells. In contrast, the lower back skin of BLM-treated TLR7-KO mice showed a statistically significant difference compared to BLM-treated WT and TLR9-KO mice.

In conclusion, the attenuation of skin and lung fibrosis caused by the TLR7 deletion was achieved through the polarized state of M2 in the spleen, followed by the migration of M2 macrophages to the target organs, ultimately inhibiting chronic inflammation and fibrosis. In contrast, a TLR9 deficiency enhanced the polarized state of M1 in the spleen, which, in turn, caused M1 macrophages to migrate to the target organs, resulting in an enhanced effect on chronic inflammation and fibrosis. Moreover, the promotion of chronic inflammation and fibrosis by the TLR9 deletion was stronger than the inhibition of chronic inflammation and fibrosis by the TLR7 deletion.

## 3. Discussion

pDCs are key cells in the immune system, particularly in antiviral responses, and they function by producing large amounts of type I interferons such as IFNα. In SSc and SLE, the activation of TLR7 and TLR9 induces the massive production of IFNα in pDCs. This process plays a central role in the pathophysiology of both diseases, as IFNα can further activate other immune cells, including APCs (antigen-presenting cells, such as M1 and M2), T cells, and B cells, which together promote inflammatory responses and autoantibody production [3,4,14,15,17,18,19]. The activation of pDCs has also been observed in SSc, and pDCs indirectly promote fibrosis by promoting T and B cell activation [2]. Our results further confirm that a TLR7 deletion leads to a balanced relationship between TLR7 and TLR9, with TLR9 predominating. This, in turn, leads to an increase in the ratio of pDCs and the production of large amounts of IFNα, indirectly inhibiting tissue fibrosis in the skin and lung tissues. Based on the result that a “TLR9 deletion hardly affects the change in the ratio of pDC”, we can conclude that TLR9 may not be a direct or critical factor in increasing the degree of skin and lung fibrosis (Figure 1 and Figure 2).

Macrophages also play an important role in the fibrotic process of SSc, particularly CD86+ (M1 type) and CD206+ (M2 type) macrophages [20]. In SLE, CD86+ (M1 type) and CD206+ (M2 type) macrophages act as APCs and play different roles in the activation of T cells and their secondary responses [21,22]. M1 macrophages tend to produce pro-inflammatory cytokines, such as TNFα and IL-6, which promote Th1 and Th17 differentiation, leading to an increase in the severity of inflammation [23]. In contrast, M2 macrophages promote tissue repair and the differentiation of Treg and Th2 cells by producing IL-10 and TGF-β [23,24,25], which was further confirmed in this study. In the absence of TLR9, TLR7 takes a dominant lead in the balance, polarizing macrophage M1 and ultimately exacerbating target organ inflammation and fibrosis. On the other hand, a TLR7 deficiency leads to the dominance of TLR9, leading to macrophage M2 polarization and, ultimately, a reduction in inflammation and fibrosis. This is achieved by enhancing the polarized state of M2 in peripheral lymphoid tissues, leading to the migration of M2 macrophages to fibrotic target organs and indirectly inhibiting the progression of inflammation by inhibiting M1 infiltration. Meanwhile, a TLR9 deletion allows TLR7 to dominate, thus enhancing the polarized state of M1 in peripheral tissues, causing M1 macrophages to migrate to fibrotic target organs, and enhancing chronic inflammation and fibrosis. Moreover, the enhancement effect of a TLR9 deletion on M1-induced chronic inflammation and fibrosis was much stronger than the inhibitory effect of M2 induced by a TLR7 deletion (Figure 6).

In SLE, immune abnormalities are associated with M1/M2 aberrations and disruptions in the balances of Th1/Th2, Breg/Beff, and Treg/Th17 [14,26,27]. The SSc experiments identified a Th1/Th2-related mechanism for the bleomycin-induced inhibition of fibrosis reduction in the skin and lung tissues of the SSc mouse model. In the case of a TLR7 deletion, it resulted in a significant reduction in the proportion of IL-4 + CD4 + Th2 cells in the spleen to achieve the suppression of chronic inflammation. In contrast, a TLR9 deficiency significantly increased the expression of TNFα + CD4 + Th1 cells in the spleen, leading to fibrosis in the skin and lung tissues, which TLR7 can achieve (Figure 5). In previous SLE studies, activated Treg and Th2 cells promoted B cell differentiation into IL-10 + CD19 + Breg cells by producing IL-10. In contrast, Th17 and Th1 cells promote B cell activation into IL-6 + CD19 + Beff cells by producing IL-17A and IFN-γ, respectively. This interaction enhances immune system activation and autoantibody production [28,29,30,31,32]. Immediately thereafter, B cells can differentiate into autoantibody-producing plasma cells by receiving signals from T cells and stimulation from other factors such as BAFF and APRIL. These autoantibodies form complexes with inflammation- and fibrosis-related antigens, leading to chronic inflammatory damage in the target organ [33,34,35,36,37]. Previous studies have shown that IL-6, a pro-inflammatory cytokine, enhances the inflammatory response and is also an important factor in skin fibrosis [38]. The SSc experiments in this study further illustrated a similar situation (Figure 3). In the SSc disease model, the absence of TLR9 led to the dominance of TLR7, which then facilitated the migration of spleen IL-6 + Beff into the skin tissue. This resulted in the release of a large amount of cytokine IL-6 and the exacerbation of the process of generating inflammatory and fibrotic responses in the skin. In contrast, IL-10 normally acts as an anti-inflammatory cytokine [39]. According to the findings of this study, the deletion of TLR7 leads to a dominant position of TLR9 in the balance, promoting an elevated proportion of IL-10 + Breg in the spleen, which then migrates to the skin tissues and inhibits IL-6 + Beff (Figure 3).

In addition, Treg (especially Foxp3 + Treg) plays a key role in maintaining immune homeostasis. However, its function may be impaired in SSc, attenuating the regulation of the fibrotic process [40,41,42]. Based on our experiments, Treg revealed a profound critical factor in maintaining immune homeostasis. The deletion of TLR7 led to the emergence of TLR9 dominance, which then indirectly enhanced the suppressive inflammatory role of Foxp3 + CD25 + CD4 + T cells in the peripheral lymphatic system. Meanwhile, the absence of TLR9 in another set of experiments attenuated the pro-inflammatory role of IL-17A + CD4 + T cells in the peripheral lymphatic system. The above suggests that, although both TLR7 and TLR9 have Treg-enhancing effects, it is TLR9 that is direct and important compared to the indirect and minor role of TLR7. Overall, there was an increased production of IL-6 and IL-10 by activated B cells (IL-6 + Beff and IL-10 + Breg, respectively), further modulating the immune response and fibrotic processes [43,44].

During fibrosis in SSc, myofibroblasts are the key executors of fibrosis, and the increased expression of α-SMA is key evidence of fibroblast-to-myofibroblast transformations [45]. Myofibroblasts produce large amounts of collagen and other components of the extracellular matrix. TGFβ and IL-6 are the key cytokines that promote this transformation [46]. Immune complex deposition in SSc is closely associated with cytokine production. Immune complexes formed by autoantibodies and antigens can be deposited in skin and lung tissues, triggering a local inflammatory response. These immune complexes can cause large secretions of cytokines that promote the progression of inflammation (e.g., TGFβ and IL-6) by activating the complementary system and attracting immune cells (e.g., macrophages and T cells) to the site of inflammation. These cytokines further activate fibroblasts and increase the expression of α-SMA and collagen, leading to tissue fibrosis [47,48,49].

Our experiments found that both TLR7 and TLR9 deletions significantly altered the outcome of tissue fibrosis. BLM-treated TLR7-KO mice had less lung and skin fibrosis than BLM-treated WT mice, whereas BLM-treated TLR9-KO mice had more lung and skin fibrosis than BLM-treated WT mice (Figure 1).

These results suggest that TLR7-TLR9 communication and balance could directly affect the balance of Breg-Beff, Treg-Th17, Th1-Th2, and M1-M2 individually and separately through pDCs (Figure 3, Figure 4, Figure 5 and Figure 6). TLR7 and TLR9, separately or simultaneously, may be potential novel therapeutic tools against SSc. However, the mechanisms by which TLR7 and TLR9 lead to immune abnormalities are complex.

This study had many limitations. Only the TLR7-KO and TLR9-KO animal models were used, and other lymphocytes downstream of the needles were analyzed. The direct interactions between TLR7 and TLR9 or the upstream correlative pathways of TLR7 and TLR9 were not analyzed. In order to further explore the interactions between TLR7 and TLR9, future studies should include a mouse disease model in which both the TLR7 and TLR9 genes are knocked out. Additionally, an in vitro cell culture and analysis at the cellular level was not performed and should be conducted in the future to provide stronger evidence for a direct association between cells in the microenvironment. Co-cultures of related lymphocytes could also be conducted to investigate the relationships between them. RT-PCR and immunohistochemistry were not included due to a shortage of mice; however, they will be conducted and analyzed in the future, as they indicate skin angiogenesis and lymphocyte infiltration.

## 4. Methods

### 4.1. Mice

WT mice were purchased from Charles River Laboratories Japan, Inc. (Kanagawa, Japan) TLR7-KO and TLR9-KO mice were purchased from OrientalBioService, Inc. (Kyoto, Japan). The mice were female, 8 to 10 weeks of age, and weighed between 18 and 22 g. They began receiving PBS and BLM treatments in the experiment. They were housed in a specific pathogen-free barrier facility and screened regularly for pathogens [43]. The Animal Experimentation Committee of the Graduate School of Medicine of Kanazawa University approved all studies and procedures.

### 4.2. Bleomycin Treatment

Bleomycin (Nippon Kayaku, Tokyo, Japan) was dissolved in PBS at a concentration of 1 mg/mL. The solution was injected intradermally (300 μL, using a 27-gauge needle) into the shaved lower back of the mice at 1-day intervals for 4 weeks from the first day, according to our previous study [50].

### 4.3. Histological Examination and IHC Analysis

A histological examination and an IHC analysis were performed on skin sections from all the mice. The sections were taken from the injected area in the middle of the lower back and extended to the body wall muscle tissue in full-thickness sections on day 28, as previously described [50]. For the lung tissue section, the entire lower lobe of the left lung was removed. Both the lung tissue and the skin section tissue were preserved in frozen paraffin blocks. The skin and lung tissue sections, each 6 mm in diameter, were stained with Masson’s trichrome stain. The dermal thickness was measured from the top of the granular layer to the junction of the dermis and intradermal fat [51]. The degree of lung fibrosis was evaluated using the Ashcroft score on the lung tissue under high magnification by two independent investigators (WC and JX) in a blinded fashion [52]. Immunohistochemical staining was performed on the cryopreserved skin tissues using antibodies against α-SMA (Sigma-Aldrich, St. Louis, MO, USA), F4/80 (Abcam, Cambridge, UK), CD86 (Serotec, Kidlington, UK), and CD206 (Santa Cruz, Santa Cruz, CA, USA). The number of stain-positive cells was counted by two independent investigators (WC and JX) in a blinded manner under high magnification.

### 4.4. RT-PCR Evaluation

After a 4-week treatment with PBS and bleomycin, we analyzed the mRNA expression levels of Il6, Il10, and Tgfb1 in the skin tissues by using quantitative real-time polymerase chain reaction (RT-PCR). The mRNA levels were normalized using GAPDH mRNA. We calculated the relative expression of the RT-PCR products using the delta–delta CT method to compare the expression of the target genes with that of the housekeeping gene GAPDH mRNA [53].

### 4.5. Flow Cytometry Analysis

Mouse spleens were removed to prepare single-cell suspensions on day 28, after the mice were treated with PBS or bleomycin. For the surface-staining experiments, coupled antibodies against PDCA-1-APC, CD11c-FITC, CD4-FITC, CD19-PE, CD25-Percp-Cy5.5, CD11b-APC-Cy7, CD86-PE-Cy7, and CD206-APC were used to stain the cell surface. The intracellular cytokine expression was observed using immunofluorescence staining and analyzed using flow cytometry. To detect IL-6, the cells (2 M cells/mL) were incubated with LPS (10 μg/mL) and anti-CD40 mAb (10 μg/mL; FGK45) for 24 h. During the last 5 h of incubation, PIB [PMA (50 ng/mL; Sigma-Aldrich), ionomycin (1 μg/mL; Sigma-Aldrich), and brefeldin A (1000× solution; BioLegend)] was added. For detecting IL-10 and IL-17A, the cells were cultured with LPS (10 μg/mL) and PIB (2 M cells/mL) for 5 h. The Fc receptor was blocked with the mouse Fc receptor mAb (2.4 G2, BD Pharmingen, San Diego, CA, USA), and dead cells were detected with a LIVE/DEAD Fixable Aqueous Dead Cell Staining Kit (Invitrogen, Waltham, MA, USA) before cell surface staining. The stained cells were fixed, permeabilized with a Cytofix/Cytoperm kit (BD Pharmingen) according to the manufacturer’s instructions, and stained for intracellular cytokines with IL-6-PE, IL-10-APC, and IL-17A-PE. The phenotype of the lymphocytes was analyzed using BD FACSCanto II (BD Biosciences, Lakes, NJ, USA). The final data were analyzed with the FlowJo software v10.8 [43].

### 4.6. Statistical Analysis

The data were shown as the mean ± SEM. An ANOVA was used for analyzing four groups of data from each experiment, followed by Tukey’s multiple comparison test. All the raw data were entered into GraphPad Prism 8.0 for an analysis and the production of statistical graphs.

## Data Availability

The data presented in this study are available on request from the corresponding author.
